# Contact chemosensation of phytochemicals by insect herbivores

**DOI:** 10.1039/c7np00002b

**Published:** 2017-05-09

**Authors:** Stefan Pentzold, Antje Burse, Wilhelm Boland

**Affiliations:** a Max Planck Institute for Chemical Ecology , Department of Bioorganic Chemistry , Hans-Knöll-Str. 8 , D-07745 Jena , Germany . Email: spentzold@ice.mpg.de ; Email: boland@ice.mpg.de

## Abstract

Contact chemosensation, or tasting, is a complex process governed by nonvolatile phytochemicals that tell host-seeking insects whether they should accept or reject a plant. During this process, insect gustatory receptors (GRs) contribute to deciphering a host plant's metabolic code. GRs recognise many different classes of nonvolatile compounds; some GRs are likely to be narrowly tuned and others, broadly tuned. Although primary and/or secondary plant metabolites influence the insect's feeding choice, their decoding by GRs is challenging, because metabolites *in planta* occur in complex mixtures that have additive or inhibitory effects; in diverse forms composed of structurally unrelated molecules; and at different concentrations depending on the plant species, its tissue and developmental stage. Future studies of the mechanism of insect herbivore GRs will benefit from functional characterisation taking into account the spatio-temporal dynamics and diversity of the plant's metabolome. Metabolic information, in turn, will help to elucidate the impact of single ligands and complex natural mixtures on the insect's feeding choice.

## Abstract 2

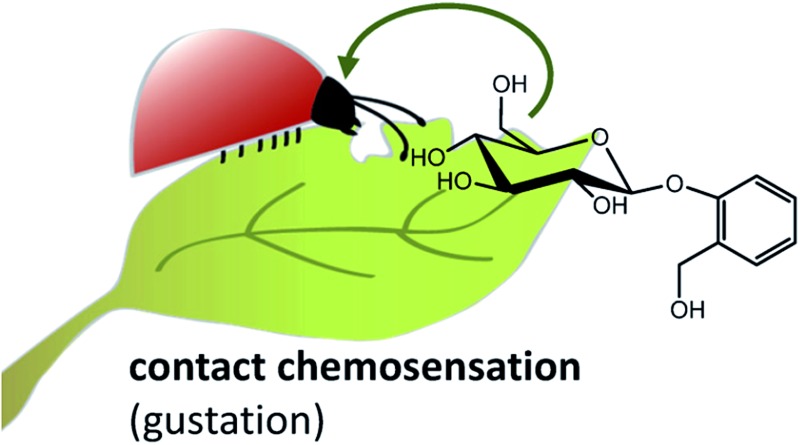
Contact chemosensation, or tasting, enables insect herbivores to identify nonvolatile metabolites in complex mixtures present in plants. The interplay of primary and secondary plant metabolites with gustatory receptors is outlined.

## 1. Introduction

All organisms are surrounded by chemical compounds in their environment; these influence many aspects of an organisms' life history. Insects possess a remarkable and complex gustatory system that enables the close-range identification of nonvolatile molecules in the highly complex mixtures that are present in plants. Such contact chemosensation (gustation, or tasting) equips insects with a final check for the suitability of hosts, mating partners and egg deposition sites.^1–3^ Because plant-feeding insects encounter a geographical and temporal mosaic of plant species, they are exposed to many different phytochemicals. In the case of specialists, only a few plant species are used as food sources; in the case of generalists, more species are consumed.^4^ To meet their nutritional requirements, insect herbivores have to integrate the nutrient content based on carbohydrates or amino acids with the often toxic content of secondary metabolites in plants. With more than 200 000 estimated compounds, the latter constitute the chemical barrier plants use to prevent herbivory and/or microbial infection.^3,5^ Consequently, the ability to recognize secondary metabolites is essential for insect herbivores. Whereas sugars or amino acids generally act as feeding stimulants, many secondary metabolites can have differing effects on insect feeding behaviour. Depending on the ability of insects to circumvent their detrimental effects, phytochemicals can act as deterrents, and also as stimulants and host-indicators.

Understanding gustatory processing is far from easy, because it involves many convergent and divergent steps: (i) the chemical composition of any plant is highly variable, due to growth characteristics, genetic variation and environmental factors, such as feeding-induced defence metabolites^5^ – consequently, the range of potential ligands for insect gustatory receptors (GRs) is highly diverse; (ii) insects possess GRs with different ligand specificities and distinct spatio-temporal expression throughout the insect's development, which often involves different hosts;^6^ (iii) insects integrate downstream signalling from GRs on a neuronal basis to higher brain centres^7^ – a process that may be influenced by dietary experience, starvation, learning and habituation.

**Fig. 1:**
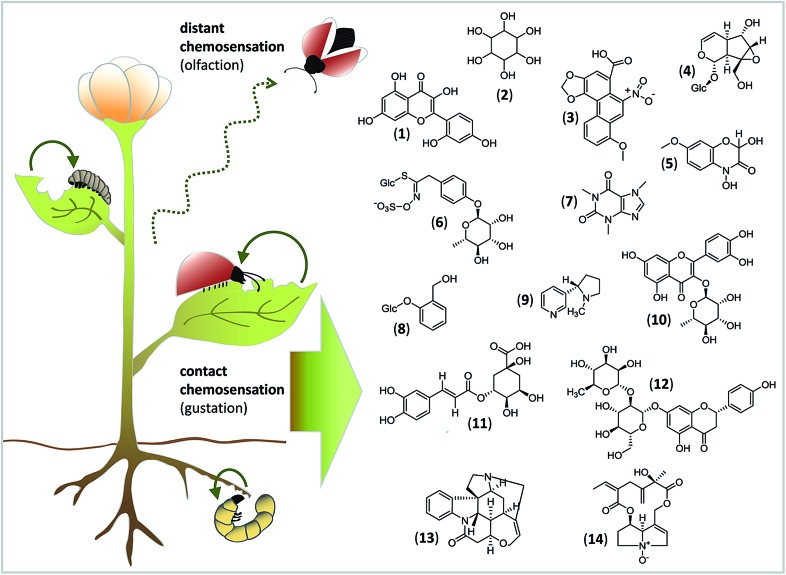
A taste of the diversity of nonvolatile phytochemicals that can be sensed by insects *via* tasting and may influence their feeding choice. Whereas distant chemosensation (dashed line) serves to detect volatile compounds for orientation, contact chemosensation (solid line) serves to detect nonvolatile compounds for the insect's final decision about the suitability of a plant as host. Compounds: (1) morin; (2) inositol; (3) aristolochic acid; (4) catalpol; (5) 2,4-dihydroxy-7-methoxy-1,4-benzoxazin-3-one (DIMBOA); (6) glucomoringin; (7) caffeine; (8) salicin; (9) nicotine; (10) quercitrin; (11) chlorogenic acid; (12) naringin; (13) strychnine; (14) seneciphylline *N*-oxide.

To give a taste of gustatory complexity, this viewpoint highlights the initial process of insect feeding and host identification from the chemical and molecular view (Fig. 1). We outline current knowledge about how different classes of nonvolatile compounds from plants are sensed by GRs of insect herbivores (for exemplary studies on gustation of *Drosophila* flies and honeybees, the reader is referred to *e.g.* ref. 7 and 8). This early phase of contact chemosensation is clearly the starting point at which natural products stimulate a response in GRs. This response is transmitted to higher brain centers and, finally, generates an adequate feeding behaviour.

## 2. GRs as mediators between phytochemicals and the insect's feeding choice

On a molecular basis, the contact chemosensation of plant chemicals and thus ligand binding occurs *via* GRs.^2^ The GR gene family has expanded in the class Insecta;^2^ whether putative homologous genes exist in other animal and plant taxa remains to be elucidated.^9^ The total number of transcripts encoding GRs within one insect herbivore species examined so far extends from 36 in adult *Phyllotreta striolata* ^10^ to 197 in *Helicoverpa armigera* ^6^ (including all developmental stages, *i.e.* egg, larva, pupa, adult male and female). This range indicates that the number of potential ligands in one plant species greatly exceeds the number of GRs in one insect herbivore species. Therefore, some GRs are likely to be narrowly tuned and others, broadly tuned. Sequence variation may have allowed fast evolutionary adaptations to detect new ligands; for example, a comparison of GRs among four lepidopteran species showed high divergence and sequence identities as small as 10%.^11^ Similarly, the GR subfamily of bitter receptors in *H. armigera* extends to 180 GRs, an expansion (in comparison to other Lepidoptera) that may be functionally linked to this insect's generalist feeding behaviour, and such a connection presumably broadens the range of secondary plant metabolites that can be detected.^6^

Among the putative GRs, only a few insect herbivore GRs have been characterised *in vitro*,^6,12–14^ which limits generalisations about their topology, ligand recognition ability and downstream signalling. Four classes of insect GR genes have been proposed to date: fructose, non-fructose, bitter/other and CO_2_ receptors.^15,16^ Insect GRs usually possess seven transmembrane domains encoded by approximately 400 amino acids with an intracellular N-terminus and extracellular C-terminus, indicating an inverted topology relative to vertebrate classical GPCRs (G protein-coupled receptors).^13,15,17^ However, a recent study suggested a variable number (three to nine) and a different orientation of transmembrane domains (both N- and C-terminus either intra- or extracellular) in the case of bitter GRs of *H. armigera*.^6^ Ligand binding on the extracellular domain initiates an intracellular signalling cascade by forming ligand-gated ion channels in a G protein-dependent or -independent manner.^12,13^ An increase of intracellular ion levels, such as of calcium, depolarises GR-containing neurons (GRNs).^12,14^ Since GRNs typically co-express many types of GRs, they can encode single ligands with unique spatio-temporal signatures, and such encoding allows the representation of these ligands in the brain.^18,19^ Usually four GRNs are housed in one taste sensillum, *i.e.* a hair-like structure with a single, terminal pore, often found on the insect's external tissues, *e.g.* palps on mouthparts, tarsi on legs, antennae and ovipositor.^20,21^ Additionally, GRs are also found in internal tissues, *e.g.* gut, brain and fat body.^17,22^ Thus, GRs and GRNs are crucial for external and internal metabolite sensation.

When an insect encounters a plant and close contact to the plant surface with the taste sensillae has been made, plant-derived ligands can diffuse into the lymph from an aqueous solution or a solid surface – a process that may be enhanced when the lymph exudes from uniporous sensillae.^3^ When an insect has started feeding, taste sensillae mainly on the mouthparts come into direct contact with the plant sap released by chewing. If compounds are water soluble, they dissolve in the lymph, whereas hydrophobic compounds may be bound by soluble binding proteins, such as chemosensory proteins, before interacting with a GR.^23^

## 3. Mixtures matter – the interplay of primary and secondary plant metabolites

Acceptance or rejection of a host plant is governed by the balance of stimulatory and deterrent compounds.^24^ Since primary and secondary metabolites in plants are found in mixtures representing many different structures (Fig. 1), concentration-dependent interactions between ligands and GRs can occur and may have additive, synergistic or inhibitory effects, and they may influence the activation of GRNs.^3^ Thus, the feeding choices of insect herbivores to mixtures can differ from their responses to single compounds. Moreover, the detection of key signals within the chemical “noise” of plant metabolite mixtures is necessary for an insect to start as well as to maintain and stop feeding.

Sugars are the main phagostimulants for insect herbivores,^3^ due to their physiological role as a universal metabolic source and their high concentration in green plants.^25^ Additionally, several studies indicate that sugars and sugar alcohols are involved in the modulation of insect responsiveness to secondary compounds. For example, sucrose significantly enhances the phagostimulatory effect of morin, a characteristic polyphenol of the mulberry leaves that are preferred by *Bombyx mori* larvae.^26^ Also, the sugar alcohol inositol enhances feeding intensity on sucrose-supplemented diets in different caterpillars.^27,28^ One of the few functional studies of a single GR using ectopic expression in insect cells and quantitative calcium imaging showed that BmGr8 from *B. mori* responds specifically to inositol.^13^ A response to inositol in *Bombyx* caterpillars occurs at 10^–3^ mM, which is far below the naturally occurring concentration of 1 mM in plants.^29^ That the combination of sugars and sugar alcohols is important for the insect's feeding choice is further illustrated by a study using *Manduca sexta*. Although caterpillars are usually deterred by noxious aristolochic acid, a solution of inositol and sucrose masks the aversive taste of aristolochic acid and renders it acceptable for feeding.^25^

Amino acids are also important phagostimulants for insects. Amino acid-detecting GRNs exhibit striking differences in sensitivity as evidenced in different lepidopteran species.^15^ That mixtures of amino acids, sucrose and secondary metabolites, *e.g.* the iridoid glycoside catalpol, can be detected by a single GRN was shown in the generalist caterpillar *Grammia geneura*.^30^ Accordingly, in feeding assays, only response-evoking compounds were phagostimulatory to *G. geneura*.^30^ On a molecular level, it remains to be elucidated if one narrow-tuned GR can detect either of these compounds, or, alternatively, if one broadly-tuned GR can detect different compound classes. For example, three different GRs in the generalist *H. armigera*, HarmGR35, 50 and 195, responded to crude extracts of cotton leaves,^6^ whereas only HarmGR195 responded to proline.^6^ However, crude extracts of tobacco, another host of *H. armigera*, did not trigger responses from HarmGR35 or HarmGR50.^6^ Assuming that both host plant species differed in more than one metabolite, these findings underpin the notion that different GRs with different ligand specificities within one insect species are responsible for decoding metabolic mixtures and thus different plant species.

## 4. Secondary plant metabolites as drivers of the insect's feeding choice

Despite the importance of primary metabolites as phagostimulants and – modulators, contact with certain secondary metabolites can also be decisive for insect feeding choice. Whereas for specialist insects, secondary metabolites often contribute to initiating and maintaining feeding, for generalist insects, these compounds may act as deterrents. Therefore, both scenarios involve the ability of the secondary metabolite(s) to generate appetitive or aversive behaviour. For example, the benzoxazinone DIMBOA (2,4-dihydroxy-7-methoxy-1,4-benzoxazin-3-one) enhances the feeding of the adapted specialist the Western corn rootworm (*Diabrotica virgifera*) on maize roots, whereas it deters the feeding of the generalist *Diabrotica balteata*.^31^ In *Pieris brassicae*, a specialist feeder on glucosinolate-containing cruciferous plants, contact with the glucosinolate glucomoringin stimulates larval feeding, which in turn elicits a response from a glucosinolate-sensitive GRN.^32^ Specialist insect herbivores often have adaptations to plant secondary metabolites, *e.g.* specific detoxification enzymes and mechanisms for sequestration, to safely handle these toxins.^4^ Thus, specialists benefit from tasting because that is how they identify competitor-free plants and sources for sequestration, whereas more generalist feeders benefit because that is how they avoid ingesting plant-derived toxins.

Compounds that taste bitter to humans, *e.g.* alkaloids, often have noxious effects. The ability to recognize bitter compounds in insects seems as important as the ability to detect sugars and seems to occur at relatively low concentrations.^15,29^ Furthermore, different bitter compounds can activate different numbers of bitter-sensitive GRNs and evoke either rejection or acceptance. Stimulation of the lateral and medial styloconic taste sensilla in specialist *Papilio hospiton* larvae with the toxins nicotine and caffeine activates all three bitter-sensitive GRNs, while stimulation with the non-toxic phenolic glycoside salicin and the flavonol glycoside quercitrin affect only two GRNs.^33^ In feeding choice assays, intact larvae ate salicin- and quercitrin-diets, but rejected nicotine and caffeine diets.^33^ Thus, the level of GRN activity may correlate with insect feeding choice, and both reflect the toxicity level of the compound sensed.

Finally, dietary experience and parasitism can change the taste perception of phytochemicals and thus feeding choice. This observation may have implications on higher trophic levels. *Pieris rapae* caterpillars reared on cabbage were strongly deterred by the phenolic chlorogenic acid, the flavanone-7-*O*-glycoside naringin and the alkaloid strychnine. However, caterpillars reared on nasturtium (*Tropaeolum majus*) did not discriminate against chlorogenic acid. It turned out that the deterrent GRN of cabbage-experienced caterpillars is more sensitive than the deterrent GRN type of nasturtium-experienced caterpillars.^34^ Infection by lethal endoparasites may alter the taste sensation of specific secondary plant metabolites, as has been shown for *G. geneura*.^35^ GRNs of parasitized caterpillars showed an increased firing rate in response to the pyrrolizidine alkaloid seneciphylline *N*-oxide and catalpol, as compared with the firing rate of unparasitized larvae. The consumption of host plants containing these compounds may increase, as the larvae try to sequester the compounds to provide a biochemical defence against the enemy.^35^ Thus, taste sensation is “optimised” to increase the insect's chemical defence, which indicates the potential involvement of GR(N)s in insect–enemy interactions.

## 5. Conclusions and future goals

Insect herbivores constitute a huge number of species, are globally distributed and can be beneficial as well as adverse for food production and human health. One requirement for their ecological success and economic importance is the ability to sense a highly diverse range of nonvolatile phytochemicals. Unlike the many morphological and physiological studies on insect herbivore gustation carried out in past decades, studies in recent years have combined state-of-the-art methods within molecular biology, analytical chemistry and bioassays, revealing first insights into the question of how single GRs function and how they may influence the insect's feeding choice. Insect taste can also be modulated by higher trophic levels, especially in the case of parasitism and the sequestration of secondary compounds used for defence. Future research on insect gustation would likely benefit from taking into account the following considerations:

• *The plant metabolome is highly dynamic*: The preformed, constitutive arsenal of plant secondary metabolites often differs in space and time within one species and among many species. Additionally, defensive compounds are rapidly induced during or after herbivory, leading to qualitative and quantitative metabolic changes. Different modes of feeding and depositing frass, oral secretions or saliva on the plant can elicit or suppress plant chemical defence.^36^ This dynamic may alter the plant's suitability as a host by increasing or decreasing toxicity, but requires that the insect sense metabolic changes.

• *Deterrence versus toxicity*: Deterrence implies that the compound is sensed and ingestion is then avoided. However, toxic compounds are not always necessarily connected to deterrence. Avoidance may be beneficial for the plant, on one hand, because post-ingestive toxic effects leading to the insect's death would stop feeding damage and tissue loss; on the other hand, avoidance puts a selection pressure on insect herbivores to tune their GRs to be able to sense the toxic compound(s).

• *GRs differ in space and time*: The spatio-temporal expression of GRs differs among tissues and developmental stages within a species.^6,37,38^ A GR expressed in the larval stage may not be expressed in the adult stage and *vice versa*, which likely reflects the variety of host plant species and thus phytochemicals encountered throughout development. For example, in many lepidopteran species, larvae are leaf-feeding, whereas adults are nectar-feeding. Furthermore, females, but not males, need to identify suitable host plants for egg deposition and are thus equipped with a GR that is likely absent in males of the same species.^20^ Whether developmental stage and diet breadth is reflected by the expression profile or ligand specificity of GRs needs to be further elucidated, mainly *via* functional characterisation.

• *Alone or in concert*: Although some *in vitro* studies indicate that single GRs function alone,^6,13,20^ it may be that an interaction with other GRs occurs *in vivo* (similar to Gr64f in *Drosophila* ^39^), or, alternatively, that a generic co-receptor modulates the activity of single GRs (similar to the odorant co-receptor Orco). Since the expression of GRs is often very low within a given tissue,^6^ proteomic profiling would be challenging but would promise to clarify whether GRs are present *in vivo* ^40^. Clearly, more GRs need to be functionally characterised in the future. However, due to the lack of information on possible ligands, especially in generalist insects, and the fact that GRs may respond to single ligands only^20^ or to aqueous extracts from one host, but not to extracts from another host,^6^ such profiling may prove difficult. An alternative is to modulate the expression of GRs *via* RNA interference (RNAi) on the post-transcriptional level or *via* CRISPR-Cas9 on the genome level. The few studies employing RNAi on insect herbivore GRs have demonstrated the involvement of a single receptor in detecting a host-specific compound.^20^ Even though functional redundancy in GRs cannot be excluded, GRs may be different in this respect in comparison to olfactory receptors. Due to the very limited knowledge on GRs that currently exists, transcript silencing or generating knockouts of GRs can be a successful way to better understand their role *in vivo*. For the latter aspect, the insect's feeding choice will need to be tested experimentally using different host plants, extracts or single compounds on a neutral substrate.^41^

Future discoveries involving insect contact chemosensation will not only improve our understanding of the fundamental question of how GRs recognize plant-derived ligands and thus how insects identify their host, but also pave the way for the use of GRs, for example, in sustainable pest management or in the fight against insect-transmitted diseases. The continuous adaptation of crop pests to insecticides requires the development of new ligands that block the specific GRs that mediate host selection and the feeding behaviour of insect herbivores.

## References

[cit1] Yarmolinsky D. A., Zuker C. S., Ryba N. J. P. (2009). Cell.

[cit2] Isono K., Morita H. (2010). Front. Cell. Neurosci..

[cit3] Chapman R. (2003). Annu. Rev. Entomol..

[cit4] Pentzold S., Zagrobelny M., Rook F., Bak S. (2014). Biol. Rev. Cambridge Philos. Soc..

[cit5] Mithöfer A., Boland W. (2012). Annu. Rev. Plant Biol..

[cit6] Xu W., Papanicolaou A., Zhang H.-J., Anderson A. (2016). Sci. Rep..

[cit7] Freeman E. G., Dahanukar A. (2015). Curr. Opin. Neurobiol..

[cit8] de Brito Sanchez M. G., Lorenzo E., Songkung S., Liu F., Zhan Y., Giurfa M. (2014). Front. Behav. Neurosci..

[cit9] Benton R. (2015). Trends Ecol. Evol..

[cit10] Wu Z., Bin S., He H., Wang Z., Li M., Lin J. (2016). PLoS One.

[cit11] Engsontia P., Sangket U., Chotigeat W., Satasook C. (2014). J. Mol. Evol..

[cit12] Sato K., Tanaka K., Touhara K. (2011). Proc. Natl. Acad. Sci. U. S. A..

[cit13] Zhang H.-J., Anderson A. R., Trowell S. C., Luo A.-R., Xiang Z.-H., Xia Q.-Y. (2011). PLoS One.

[cit14] Jiang X.-J., Ning C., Guo H., Jia Y.-Y., Huang L.-Q., Qu M.-J., Wang C.-Z. (2015). Insect Biochem. Mol. Biol..

[cit15] Agnihotri A., Roy A., Joshi R. (2016). Insect Mol. Biol..

[cit16] Xu W., Anderson A. (2015). Sci. Nat..

[cit17] Xu W., Zhang H.-J., Anderson A. (2012). J. Chem. Ecol..

[cit18] Wright G. A. (2016). Curr. Opin. Neurobiol..

[cit19] Reiter S., Rodriguez C. C., Sun K., Stopfer M. (2015). J. Neurosci..

[cit20] Ozaki K., Ryuda M., Yamada A., Utoguchi A., Ishimoto H., Calas D., Marion-Poll F., Tanimura T., Yoshikawa H. (2011). Nat. Commun..

[cit21] Seada M. A., Ignell R., Anderson P. (2016). J. Entomol. Sci..

[cit22] Koenig C., Hirsh A., Bucks S., Klinner C., Vogel H., Shukla A., Mansfield J. H., Morton B., Hansson B. S., Grosse-Wilde E. (2015). Insect Biochem. Mol. Biol..

[cit23] Pelosi P., Iovinella I., Felicioli A., Dani F. R. (2014). Front. Physiol..

[cit24] Sollai G., Biolchini M., Solari P., Crnjar R. (2017). J. Insect Physiol..

[cit25] Cocco N., Glendinning J. I. (2012). J. Exp. Biol..

[cit26] Hamamura Y., Hayashiya K., Naito K.-I., Matsuura K., Nishida J. (1962). Nature.

[cit27] Sasaki K. E. N., Ooki Y., Endo Y., Asaoka K. (2013). Physiol. Entomol..

[cit28] Glendinning J. I., Jerud A., Reinherz A. T. (2007). J. Exp. Biol..

[cit29] Zhang H.-J., Faucher C. P., Anderson A., Berna A. Z., Trowell S., Chen Q.-M., Xia Q.-Y., Chyb S. (2013). J. Chem. Ecol..

[cit30] Bernays E. A., Chapman R. F., Singer M. S. (2000). J. Comp. Physiol., A.

[cit31] Robert C. A. M., Veyrat N., Glauser G., Marti G., Doyen G. R., Villard N., Gaillard M. D. P., Köllner T. G., Giron D., Body M. (2012). Ecol. Lett..

[cit32] Müller C., van Loon J., Ruschioni S., De Nicola G. R., Olsen C. E., Iori R., Agerbirk N. (2015). Phytochemistry.

[cit33] Sollai G., Barbarossa I. T., Solari P., Crnjar R. (2015). J. Insect Physiol..

[cit34] Zhou D., Wang C.-Z., Van Loon J. (2009). J. Insect Physiol..

[cit35] Bernays E. A., Singer M. S. (2005). Nature.

[cit36] Ray S., Basu S., Rivera-Vega L. J., Acevedo F. E., Louis J., Felton G. W., Luthe D. S. (2016). J. Chem. Ecol..

[cit37] Dippel S., Kollmann M., Oberhofer G., Montino A., Knoll C., Krala M., Rexer K.-H., Frank S., Kumpf R., Schachtner J. (2016). BMC Biol..

[cit38] Guo H., Cheng T., Chen Z., Jiang L., Guo Y., Liu J., Li S., Taniai K., Asaoka K., Kadono-Okuda K. (2017). Insect Biochem. Mol. Biol..

[cit39] Jiao Y., Moon S. J., Wang X., Ren Q., Montell C. (2008). Curr. Biol..

[cit40] Alabi T., Marion-Poll F., Danho M., Mazzucchelli G., De Pauw E., Haubruge E., Francis F. (2014). Insect Mol. Biol..

[cit41] Müller C., Renwick J. A. A. (2001). Chemoecology.

